# The genome sequence of a hoverfly, *Epistrophe eligans *(Harris, 1780)

**DOI:** 10.12688/wellcomeopenres.20336.1

**Published:** 2023-11-13

**Authors:** Olga Sivell, Duncan Sivell, Liam M. Crowley, Steven Falk, Natural History Museum Genome Acquisition Lab, University of Oxford and Wytham Woods Genome Acquisition Lab, Darwin Tree of Life Barcoding collective, Wellcome Sanger Institute Tree of Life programme, Wellcome Sanger Institute Scientific Operations: DNA Pipelines collective, Tree of Life Core Informatics collective, Darwin Tree of Life Consortium

**Affiliations:** 1 Natural History Museum, London, England, UK; 2 University of Oxford, Oxford, England, UK; 3 Independent researcher, Kenilworth, England, UK

**Keywords:** Epistrophe eligans, Spring Epistrophe, genome sequence, chromosomal, Diptera

## Abstract

We present a genome assembly from an individual female *Epistrophe eligans* (the Spring Epistrophe; Arthropoda; Insecta; Diptera; Syrphidae). The genome sequence is 405.9 megabases in span. Most of the assembly is scaffolded into 5 chromosomal pseudomolecules, including the X sex chromosome. The mitochondrial genome has also been assembled and is 16.93 kilobases in length.

## Species taxonomy

Eukaryota; Metazoa; Eumetazoa; Bilateria; Protostomia; Ecdysozoa; Panarthropoda; Arthropoda; Mandibulata; Pancrustacea; Hexapoda; Insecta; Dicondylia; Pterygota; Neoptera; Endopterygota; Diptera; Brachycera; Muscomorpha; Eremoneura; Cyclorrhapha; Aschiza; Syrphoidea; Syrphidae; Syrphinae; Syrphini; *Epistrophe*; *Epistrophe eligans* (Harris, 1780) (NCBI:txid1124511).

## Background

*Epistrophe eligans* is a common bee-mimicking hoverfly (Syrphidae) with a dark bronze thorax, yellow scutellum and variable yellow markings on a dark abdomen. It is a medium-sized species with a body length of 10–11 mm ( van Veen, 2014) and a wing length 6.25–9.5 mm ( Ball & Morris, 2015). It is one of seven species in the genus *Epistrophe* occurring in Britain ( Chandler, 2023). It can be distinguished from the other species in the genus by having reduced yellow markings (spots or bands) on abdominal tergite four as compared to tergite three, or tergite four may be completely dark; tergite 2 has two spots, wedges or narrowly separated bars ( Ball & Morris, 2015; van Veen, 2014).

The larvae of *Epistrophe eligans* are an elongated oval shape, dorsoventrally flattened, green with a medial white stripe on their dorsal surface and white, orange and green flecks. They turn pale brown when in diapause. They are primarily nocturnal, although starving individuals may be active during the day. During the day they rest concealed on the underside of open leaves and do not enter leaf folds ( Rotheray, 1986). The larvae can be found on trees and shrubs especially fruit trees, elder *Sambucus nigra*, sycamore *Acer pseudoplanatus* and bramble *Rubus fruticosus*, where they feed on aphids. *Epistrophe eligans* adults can be encountered on a variety of flowers in spring, particularly of trees and bushes such as blackthorn *Prunus spinosa* and hawthorn *Crataegus monogyna* ( Ball & Morris, 2015; Stubbs & Falk, 2002).

*Epistrophe eligans* is widely distributed across Europe, extending east to the Caucasus ( van Veen, 2014). This species is common in southern England and Wales, reaching northern England and central and coastal Scotland, but it is less common in the north although there is recent evidence of a northward expansion in range ( Ball *et al.*, 2011; Ball & Morris, 2015). Adults can be found near bushes and trees, particularly in woodland rides and on sunny wood edges, and are on the wing from March to August, although the majority of records are in April and May ( Ball *et al*., 2011). In recent years the males have been emerging earlier due to warmer springs, from late March in south-east England ( Ball & Morris, 2015; Stubbs & Falk, 2002).

The high-quality genome of *Epistrophe eligans* was sequenced as part of the Darwin Tree of Life Project, a collaborative effort to sequence all named eukaryotic species in the Atlantic Archipelago of Britain and Ireland. We hope these data will support research into taxonomy, biology, phenology and phylogeny of the species and the group and effects of climate change on insect communities. Here we present a chromosomally complete genome sequence for *Epistrophe eligans*, based on a female specimen from Luton.

## Genome sequence report

**Figure 1.**
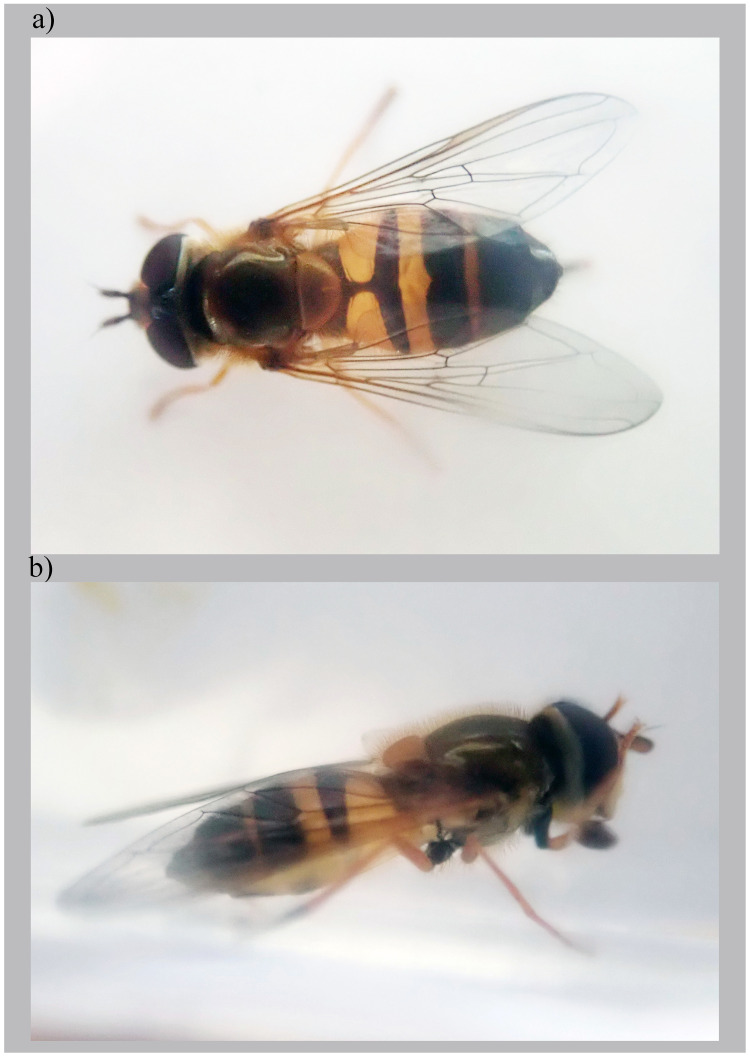
*Epistrophe eligans* (Harris, 1780), female. The specimen (NHMUK014111051; idEpiElig2) used for genome sequencing: **a**) dorsal view, **b**) lateral view.

The genome was sequenced from one female *Epistrophe eligans* ( Figure 1) collected from Luton, UK (51.89, –0.39). A total of 50-fold coverage in Pacific Biosciences single-molecule HiFi long reads was generated. Primary assembly contigs were scaffolded with chromosome conformation Hi-C data. Manual assembly curation corrected 29 missing joins or mis-joins and removed one haplotypic duplication, reducing the assembly length by 0.16% and the scaffold number by 10.79%, and increasing the scaffold N50 by 11.19%.

**Figure 2.**
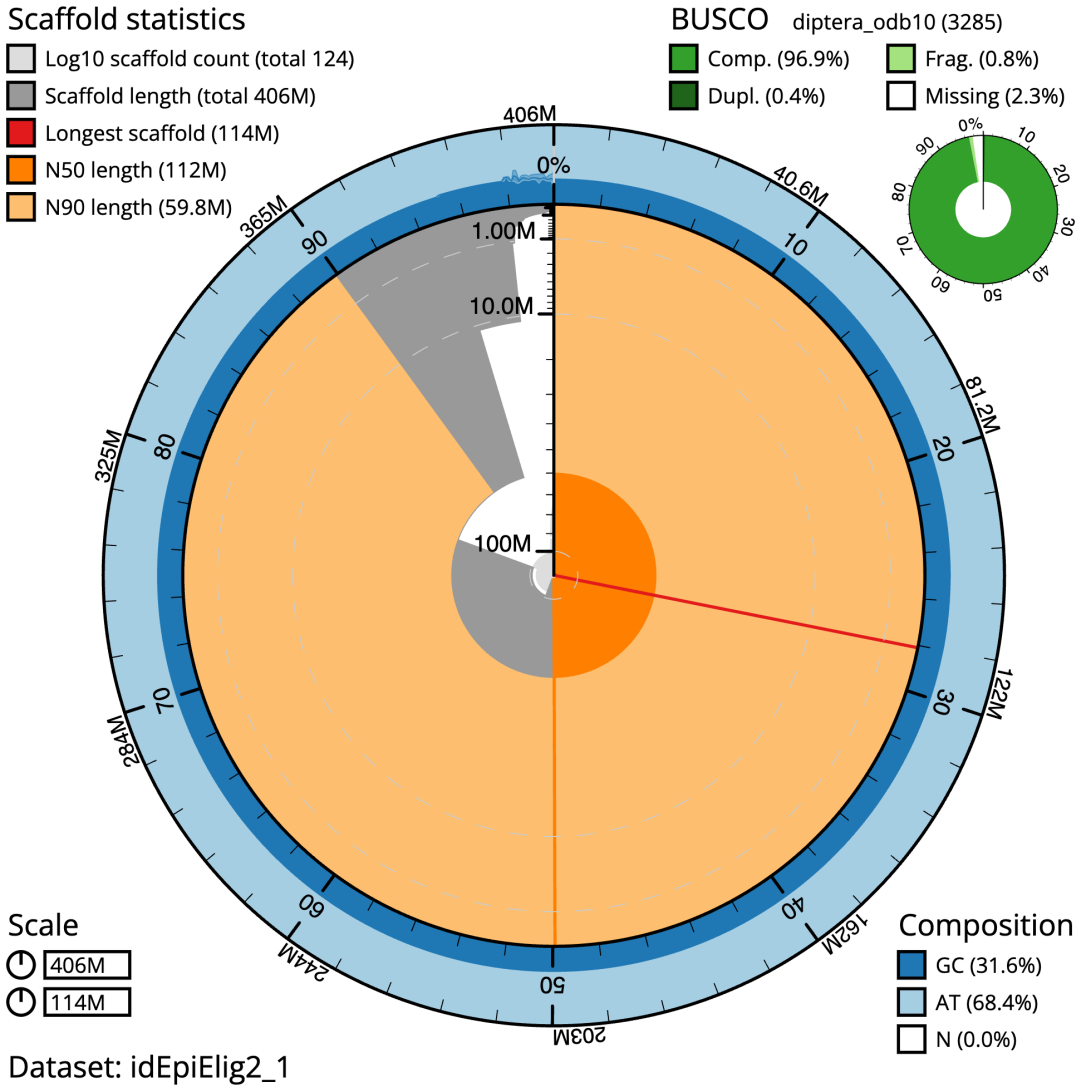
Genome assembly of *Epistrophe eligans*, idEpiElig2.1: metrics. The BlobToolKit Snailplot shows N50 metrics and BUSCO gene completeness. The main plot is divided into 1,000 size-ordered bins around the circumference with each bin representing 0.1% of the 405,900,150 bp assembly. The distribution of scaffold lengths is shown in dark grey with the plot radius scaled to the longest scaffold present in the assembly (114,255,398 bp, shown in red). Orange and pale-orange arcs show the N50 and N90 scaffold lengths (111,598,366 and 59,794,574 bp), respectively. The pale grey spiral shows the cumulative scaffold count on a log scale with white scale lines showing successive orders of magnitude. The blue and pale-blue area around the outside of the plot shows the distribution of GC, AT and N percentages in the same bins as the inner plot. A summary of complete, fragmented, duplicated and missing BUSCO genes in the diptera_odb10 set is shown in the top right. An interactive version of this figure is available at https://blobtoolkit.genomehubs.org/view/Epistrophe%20eligans/dataset/idEpiElig2_1/snail.

**Figure 3.**
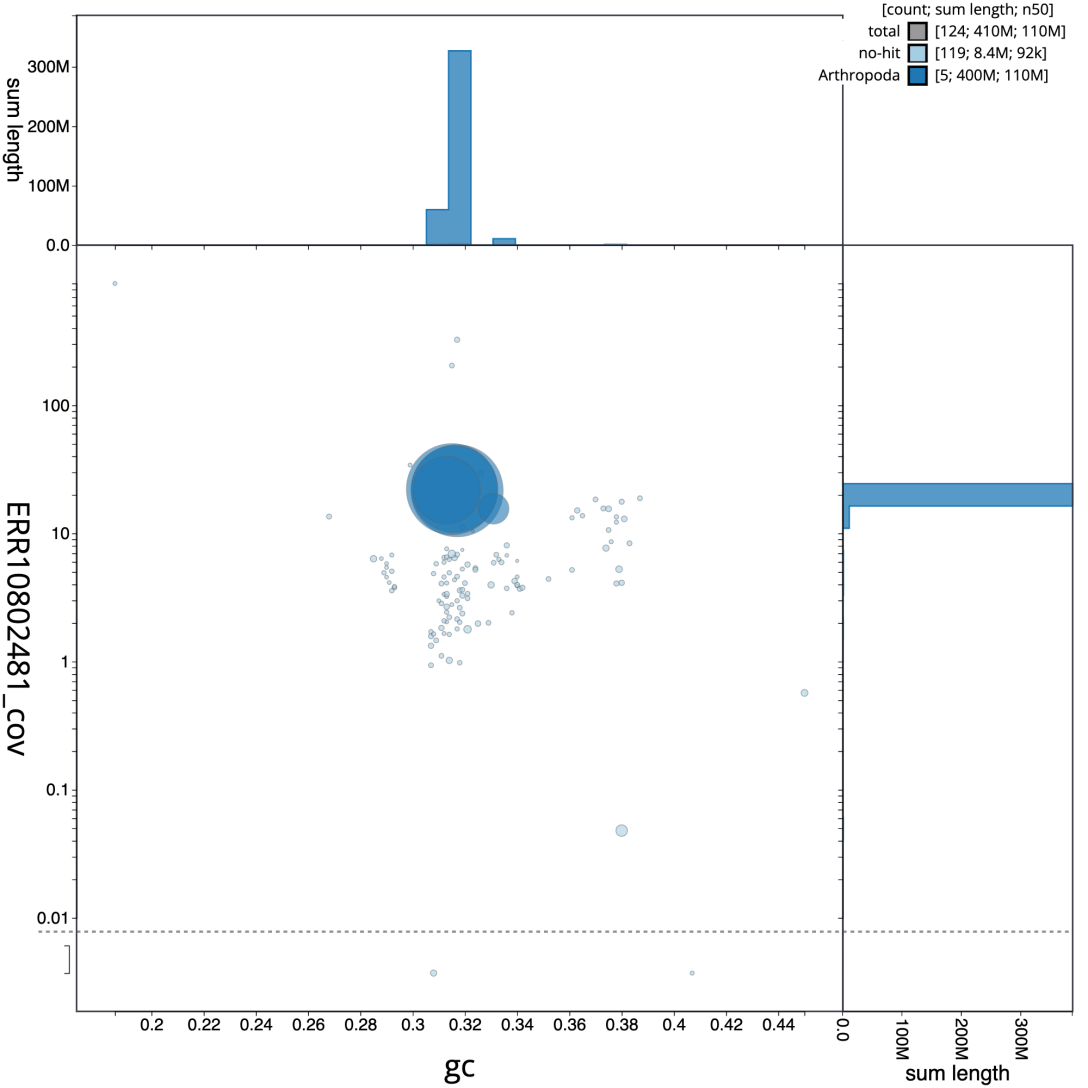
Genome assembly of *Epistrophe eligans*, idEpiElig2.1: BlobToolKit GC-coverage plot. Scaffolds are coloured by phylum. Circles are sized in proportion to scaffold length. Histograms show the distribution of scaffold length sum along each axis. An interactive version of this figure is available at https://blobtoolkit.genomehubs.org/view/Epistrophe%20eligans/dataset/idEpiElig2_1/blob.

**Figure 4.**
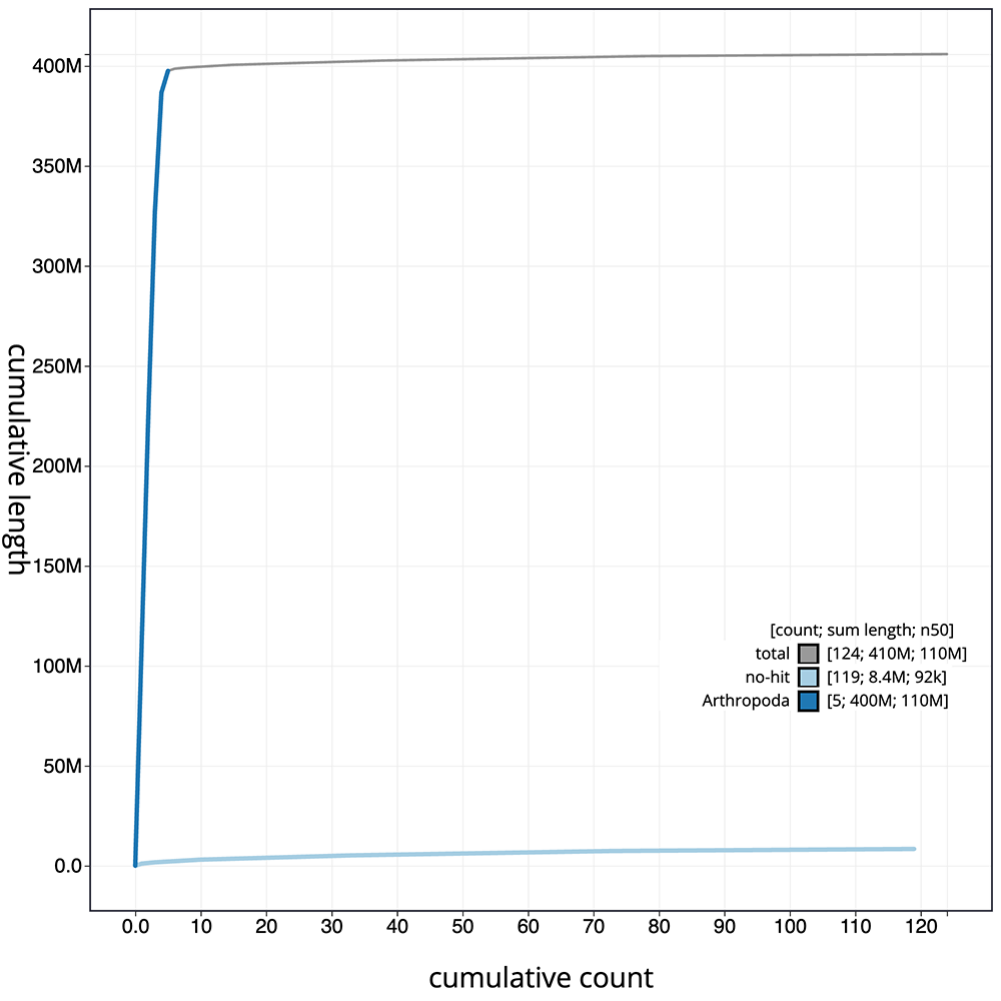
Genome assembly of *Epistrophe eligans*, idEpiElig2.1: BlobToolKit cumulative sequence plot. The grey line shows cumulative length for all scaffolds. Coloured lines show cumulative lengths of scaffolds assigned to each phylum using the buscogenes taxrule. An interactive version of this figure is available at https://blobtoolkit.genomehubs.org/view/Epistrophe%20eligans/dataset/idEpiElig2_1/cumulative.

**Figure 5.**
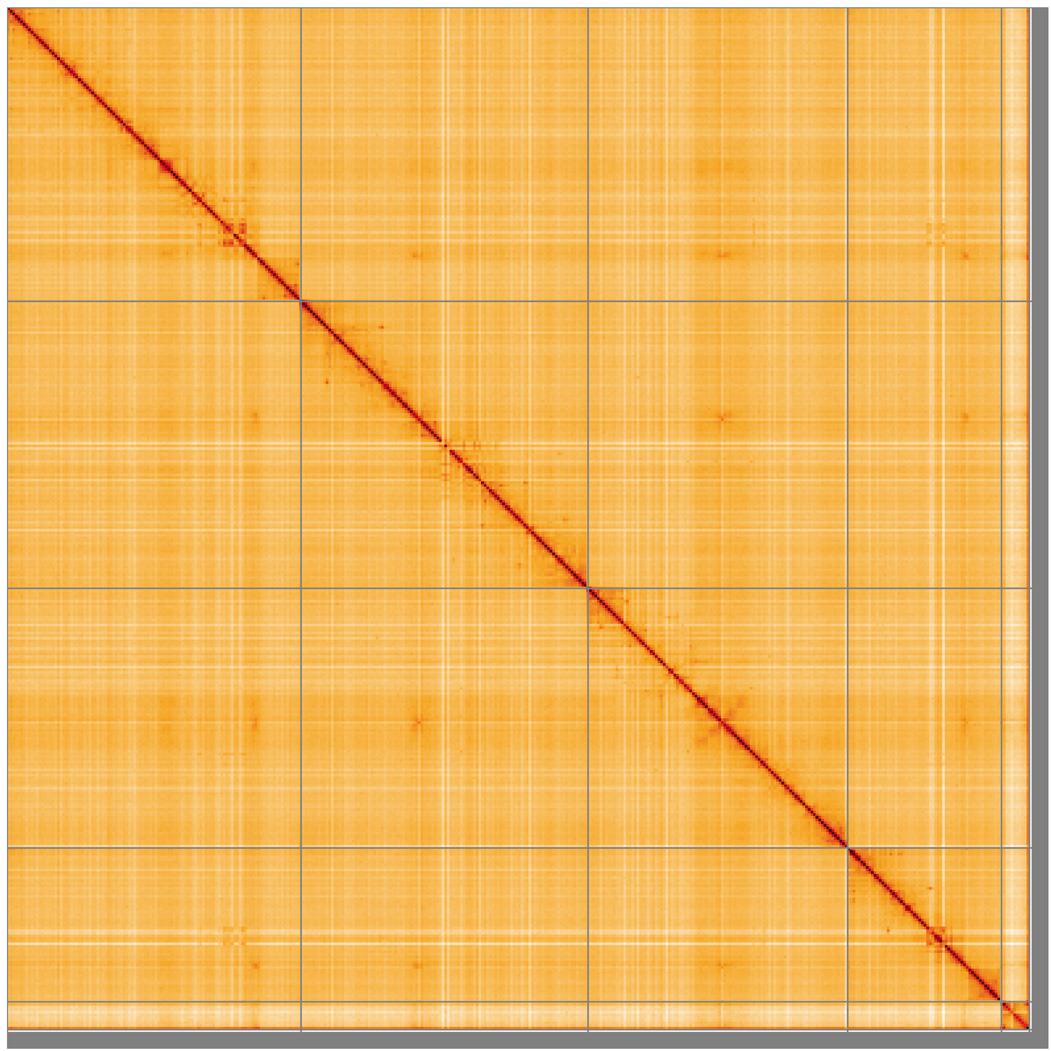
Genome assembly of *Epistrophe eligans*, idEpiElig2.1: Hi-C contact map of the idEpiElig2.1 assembly, visualised using HiGlass. Chromosomes are shown in order of size from left to right and top to bottom. An interactive version of this figure may be viewed at https://genome-note-higlass.tol.sanger.ac.uk/l/?d=A0S9vA7YQVWF58hnGPJ1xA.

The final assembly has a total length of 405.9 Mb in 123 sequence scaffolds with a scaffold N50 of 111.6 Mb ( Table 1). The snailplot in Figure 2 provides a summary of the assembly statistics, while the distribution of assembly scaffolds on GC proportion and coverage is shown in Figure 3. The cumulative assembly plot in Figure 4 shows curves for subsets of scaffolds assigned to different phyla. Most (97.95%) of the assembly sequence was assigned to 5 chromosomal-level scaffolds, representing 4 autosomes and the X sex chromosome. Chromosome-scale scaffolds confirmed by the Hi-C data are named in order of size ( Figure 5; Table 2). Chromosome X was identified by synteny to *Epistrophe grossulariae (*GCA_929447395.1) ( Falk *et al.*, 2023). While not fully phased, the assembly deposited is of one haplotype. Contigs corresponding to the second haplotype have also been deposited. The mitochondrial genome was also assembled and can be found as a contig within the multifasta file of the genome submission.

**Table 1.** Genome data for *Epistrophe eligans*, idEpiElig2.1.

Project accession data		
Assembly identifier	idEpiElig2.1	
Assembly release date	2023-05-13	
Species	*Epistrophe eligans*	
Specimen	idEpiElig2	
NCBI taxonomy ID	1124511	
BioProject	PRJEB59159	
BioSample ID	SAMEA7521388	
Isolate information	idEpiElig2: DNA idEpiElig1: Hi-C	
Assembly metrics *		*Benchmark*
Consensus quality (QV)	62	*≥ 50*
*k*-mer completeness	100%	*≥ 95%*
BUSCO **	C:96.9%[S:96.5%,D:0.4%], F:0.8%,M:2.3%,n:3,285	*C ≥ 95%*
Percentage of assembly mapped to chromosomes	97.95%	*≥ 95%*
Sex chromosomes	X chromosome	*localised * *homologous pairs*
Organelles	Mitochondrial genome assembled	*complete single * *alleles*
Raw data accessions		
PacificBiosciences SEQUEL II	ERR10809398	
Hi-C Illumina	ERR10802480	
PolyA RNA-Seq Illumina	ERR10908616	
Genome assembly		
Assembly accession	GCA_951394125.1	
*Accession of alternate haplotype*	GCA_951394095.1	
Span (Mb)	405.9	
Number of contigs	248	
Contig N50 length (Mb)	5.8	
Number of scaffolds	123	
Scaffold N50 length (Mb)	111.6	
Longest scaffold (Mb)	114.3	

* Assembly metric benchmarks are adapted from column VGP-2020 of “Table 1: Proposed standards and metrics for defining genome assembly quality” from ( Rhie *et al.*, 2021). ** BUSCO scores based on the diptera_odb10 BUSCO set using v5.3.2. C = complete [S = single copy, D = duplicated], F = fragmented, M = missing, n = number of orthologues in comparison. A full set of BUSCO scores is available at https://blobtoolkit.genomehubs.org/view/Epistrophe%20eligans/dataset/idEpiElig2_1/busco.

**Table 2.** Chromosomal pseudomolecules in the genome assembly of *Epistrophe eligans*, idEpiElig2.

INSDC accession	Chromosome	Length (Mb)	GC%
OX596028.1	1	114.26	31.5
OX596029.1	2	111.6	31.5
OX596030.1	3	100.96	31.5
OX596031.1	4	59.79	31.5
OX596032.1	X	10.9	33.0
OX596033.1	MT	0.02	18.5

The estimated Quality Value (QV) of the final assembly is 62 with *k*-mer completeness of 100%, and the assembly has a BUSCO v5.3.2 completeness of 96.9% (single = 96.5%, duplicated = 0.4%), using the diptera_odb10 reference set ( *n* = 3,285).

Metadata for specimens, barcode results, spectra estimates, sequencing runs, contaminants and pre-curation assembly statistics are given at https://links.tol.sanger.ac.uk/species/1124511.

## Methods

### Sample acquisition and nucleic acid extraction

The specimen used for genome sequencing was a female *Epistrophe eligans* (specimen ID NHMUK014111051, ToLID idEpiElig2), netted in an urban garden in Luton, UK (latitude 51.89, longitude –0.39) on 2020-06-16. The specimen was collected by Olga Sivell (Natural History Museum) and identified by Duncan Sivell (Natural History Museum) and preserved on dry ice.

The specimen used for Hi-C sequencing (specimen ID Ox000429, ToLID idEpiElig1) was netted in Wytham Woods, Oxfordshire (biological vice-county Berkshire), UK (latitude 51.77, longitude –1.34) on 2020-05-22. The specimen was collected and identified by Liam Crowley (University of Oxford). A third specimen (specimen ID Ox001231, ToLID idEpiElig3), collected in Wytham Woods by Steven Falk (independent researcher) on 2021-04-19, was used for RNA sequencing.

High molecular weight (HMW) DNA was extracted at the Tree of Life laboratory, Wellcome Sanger Institute (WSI), following a sequence of core procedures: sample preparation; sample homogenisation; HMW DNA extraction; DNA fragmentation; and DNA clean-up. The idEpiElig2 sample was weighed and dissected on dry ice (as per the protocol https://dx.doi.org/10.17504/protocols.io.x54v9prmqg3e/v1). The thorax of the idEpiElig2 sample was homogenised using a Nippi Powermasher fitted with a BioMasher pestle, following the protocol at https://dx.doi.org/10.17504/protocols.io.5qpvo3r19v4o/v1. DNA was extracted by means of the HMW DNA Extraction: Automated MagAttract protocol ( https://dx.doi.org/10.17504/protocols.io.kxygx3y4dg8j/v1). HMW DNA was sheared into an average fragment size of 12–20 kb in a Megaruptor 3 system with speed setting 30, following the HMW DNA Fragmentation: Diagenode Megaruptor®3 for PacBio HiFi protocol ( https://dx.doi.org/10.17504/protocols.io.8epv5x2zjg1b/v1). Sheared DNA was purified using Manual solid-phase reversible immobilisation (SPRI) (protocol at https://dx.doi.org/10.17504/protocols.io.kxygx3y1dg8j/v1). In brief, the method employs a 1.8X ratio of AMPure PB beads to sample to eliminate shorter fragments and concentrate the DNA. The concentration of the sheared and purified DNA was assessed using a Nanodrop spectrophotometer and Qubit Fluorometer and Qubit dsDNA High Sensitivity Assay kit. Fragment size distribution was evaluated by running the sample on the FemtoPulse system.

RNA was extracted from abdomen tissue of idEpiElig3 using the Automated MagMax™ *mir*Vana protocol ( https://dx.doi.org/10.17504/protocols.io.6qpvr36n3vmk/v1). The RNA concentration was assessed using a Nanodrop spectrophotometer and Qubit Fluorometer using the Qubit RNA Broad-Range (BR) Assay kit. Analysis of the integrity of the RNA was done using the Agilent RNA 6000 Pico Kit and Eukaryotic Total RNA assay.

All wet lab protocols developed by the Tree of Life laboratory are publicly available on protocols.io: https://dx.doi.org/10.17504/protocols.io.8epv5xxy6g1b/v1.

### Sequencing

Pacific Biosciences HiFi circular consensus DNA sequencing libraries were constructed according to the manufacturers’ instructions. Poly(A) RNA-Seq libraries were constructed using the NEB Ultra II RNA Library Prep kit. DNA and RNA sequencing was performed by the Scientific Operations core at the WSI on Pacific Biosciences SEQUEL II (HiFi) and Illumina NovaSeq 6000 (RNA-Seq) instruments. Hi-C data were also generated from head and thorax tissue of idEpiElig1 using the Arima2 kit and sequenced on the HiSeq X Ten instrument.

### Genome assembly, curation and evaluation

Assembly was carried out with Hifiasm ( Cheng *et al.*, 2021) and haplotypic duplication was identified and removed with purge_dups ( Guan *et al.*, 2020). The assembly was then scaffolded with Hi-C data ( Rao *et al.*, 2014) using YaHS ( Zhou *et al.*, 2023). The assembly was checked for contamination and corrected as described previously ( Howe *et al.*, 2021). Manual curation was performed using HiGlass ( Kerpedjiev *et al.*, 2018) and Pretext ( Harry, 2022). The mitochondrial genome was assembled using MitoHiFi ( Uliano-Silva *et al.*, 2023), which runs MitoFinder ( Allio *et al.*, 2020) or MITOS ( Bernt *et al.*, 2013) and uses these annotations to select the final mitochondrial contig and to ensure the general quality of the sequence.

A Hi-C map for the final assembly was produced using bwa-mem2 ( Vasimuddin *et al.*, 2019) in the Cooler file format ( Abdennur & Mirny, 2020). To assess the assembly metrics, the *k*-mer completeness and QV consensus quality values were calculated in Merqury ( Rhie *et al.*, 2020). This work was done using Nextflow ( Di Tommaso *et al.*, 2017) DSL2 pipelines “sanger-tol/readmapping” ( Surana *et al.*, 2023a) and “sanger-tol/genomenote” ( Surana *et al.*, 2023b). The genome was analysed within the BlobToolKit environment ( Challis *et al.*, 2020) and BUSCO scores ( Manni *et al.*, 2021; Simão *et al.*, 2015) were calculated.

Table 3 contains a list of relevant software tool versions and sources.

**Table 3.** Software tools: versions and sources.

Software tool	Version	Source
BlobToolKit	4.2.1	https://github.com/blobtoolkit/blobtoolkit
BUSCO	5.3.2	https://gitlab.com/ezlab/busco
Hifiasm	0.16.1-r375	https://github.com/chhylp123/hifiasm
HiGlass	1.11.6	https://github.com/higlass/higlass
Merqury	MerquryFK	https://github.com/thegenemyers/MERQURY.FK
MitoHiFi	2	https://github.com/marcelauliano/MitoHiFi
PretextView	0.2	https://github.com/wtsi-hpag/PretextView
purge_dups	1.2.3	https://github.com/dfguan/purge_dups
sanger-tol/genomenote	v1.0	https://github.com/sanger-tol/genomenote
sanger-tol/readmapping	1.1.0	https://github.com/sanger-tol/readmapping/tree/1.1.0
YaHS	1.2a	https://github.com/c-zhou/yahs

### Wellcome Sanger Institute – Legal and Governance

The materials that have contributed to this genome note have been supplied by a Darwin Tree of Life Partner. The submission of materials by a Darwin Tree of Life Partner is subject to the **‘Darwin Tree of Life Project Sampling Code of Practice’**, which can be found in full on the Darwin Tree of Life website here. By agreeing with and signing up to the Sampling Code of Practice, the Darwin Tree of Life Partner agrees they will meet the legal and ethical requirements and standards set out within this document in respect of all samples acquired for, and supplied to, the Darwin Tree of Life Project. 

Further, the Wellcome Sanger Institute employs a process whereby due diligence is carried out proportionate to the nature of the materials themselves, and the circumstances under which they have been/are to be collected and provided for use. The purpose of this is to address and mitigate any potential legal and/or ethical implications of receipt and use of the materials as part of the research project, and to ensure that in doing so we align with best practice wherever possible. The overarching areas of consideration are:

•     Ethical review of provenance and sourcing of the material

•     Legality of collection, transfer and use (national and international) 

Each transfer of samples is further undertaken according to a Research Collaboration Agreement or Material Transfer Agreement entered into by the Darwin Tree of Life Partner, Genome Research Limited (operating as the Wellcome Sanger Institute), and in some circumstances other Darwin Tree of Life collaborators.

## Data Availability

European Nucleotide Archive: *Epistrophe eligans* (spring epistrophe). Accession number PRJEB59159; https://identifiers.org/ena.embl/PRJEB59159 ( Wellcome Sanger Institute, 2023). The genome sequence is released openly for reuse. The *Epistrophe eligans* genome sequencing initiative is part of the Darwin Tree of Life (DToL) project. All raw sequence data and the assembly have been deposited in INSDC databases. The genome will be annotated using available RNA-Seq data and presented through the Ensembl pipeline at the European Bioinformatics Institute. Raw data and assembly accession identifiers are reported in Table 1.

## Author information

Members of the University of Oxford and Wytham Woods Genome Acquisition Lab are listed here: https://doi.org/10.5281/zenodo.7125292.

Members of the Natural History Museum Genome Acquisition Lab are listed here: https://doi.org/10.5281/zenodo.7139035.

Members of the Darwin Tree of Life Barcoding collective are listed here: https://doi.org/10.5281/zenodo.4893703.

Members of the Wellcome Sanger Institute Tree of Life programme are listed here: https://doi.org/10.5281/zenodo.4783585.

Members of Wellcome Sanger Institute Scientific Operations: DNA Pipelines collective are listed here: https://doi.org/10.5281/zenodo.4790455.

Members of the Tree of Life Core Informatics collective are listed here: https://doi.org/10.5281/zenodo.5013541.

Members of the Darwin Tree of Life Consortium are listed here: https://doi.org/10.5281/zenodo.4783558.
