# Supplementary material for: The genome sequence of a hoverfly, Epistrophe eligans (Harris, 1780)
Source: Wellcome Open Res. 2023 Nov 13;8:521. [Version 1] doi: 10.12688/wellcomeopenres.20336.1 (PMC11362734; doi:10.12688/wellcomeopenres.20336.1)
Supplement: Peer review history

# Reviewer report: Reviewer response for version 1

**DOI:** 10.21956/wellcomeopenres.22519.r71213

**Published:** 2024-08-29

**Authors:** Luis Diambra

**Affiliations:** 1 National University of La Plata, Argentina, Argentina

This manuscript written by Olga Sivell et al. presents a high-quality genome of Diptera *Epistrophe eligans*, together with the RNAseq and Hi-C data. The data generation and analysis process is comprehensively clarified and the result provides ample information to understand this important genomic asset. Overall, this paper exhibits a commendable standard and deserves Indexing in the journal.

 I just want to point out two minor points:

- I find some redundancy between Figs. 2 and 3, in any case, the latter is very little commented on in the text to understand what its contribution is.
- On the other hand, authors could add the chromosome labels to the margin of Fig. 5.

Are sufficient details of methods and materials provided to allow replication by others?

Yes

Is the rationale for creating the dataset(s) clearly described?

Yes

Are the datasets clearly presented in a useable and accessible format?

Yes

Are the protocols appropriate and is the work technically sound?

Yes

Reviewer Expertise:

Bioinformatics

I confirm that I have read this submission and believe that I have an appropriate level of expertise to confirm that it is of an acceptable scientific standard.

# Reviewer report: Reviewer response for version 1

**DOI:** 10.21956/wellcomeopenres.22519.r71212

**Published:** 2024-01-19

**Authors:** Jaakko Pohjoismäki

**Affiliations:** 1 Department of Environmental and Biological Sciences, University of Eastern Finland, Joensuu, Finland

Olga Sivell, Duncan Sivell, Liam Crowley, Steven Falk and the DToL consortium present a chromosome-level genome assembly of a female *Epistrophe eligans*. It is a nice addition to the growing number of Brachyceran genomes.

 The background part gives a sufficient introduction to the species, methods, data presentation and the genome quality follow the usual DToL standards. Overall, the report is generally well written.

 For a member of the genus, the species is very easy to identify and the presented photos leave no doubt also of the sex of the specimen. Some mention of the sexual dimorphism in the species might be a good addition to the background? I have to wonder why a female was chosen over the male (idEpiElig1) specimen, which would have been the heterogametic sex? Evolution of sex chromosomes is a fascinating topic and obtaining the complete sequences of both sex chromosomes - where possible - would be valuable for future research.

 Also, although a link to the specimen metadata, barcode results, spectra estimates, sequencing runs, contaminants and pre-curation assembly statistics etc is provided, these could have been briefly described in the report. For example, there is a whole section of cobionts, which are highly interesting. How were these identified? Also while *Pseudomonas *is probably a common contaminant, the specialised endosymbionts could have been mentioned, as this would make them easier to discover in the future when scrolling through the genome reports. I also don't see any DNA barcoding results mentioned in the report or presented under the given link? I hope it will not be a big deal to add these still?

 Despite the small criticism above, the continuing effort to chart genomes of also the much neglected parts the European biodiversity is highly appreciated.

Are sufficient details of methods and materials provided to allow replication by others?

Partly

Is the rationale for creating the dataset(s) clearly described?

Yes

Are the datasets clearly presented in a useable and accessible format?

Yes

Are the protocols appropriate and is the work technically sound?

Yes

Reviewer Expertise:

Diptera taxonomy, DNA barcoding, mitochondria, cell and molecular biology

I confirm that I have read this submission and believe that I have an appropriate level of expertise to confirm that it is of an acceptable scientific standard, however I have significant reservations, as outlined above.

# Reviewer report: Reviewer response for version 1

**DOI:** 10.21956/wellcomeopenres.22519.r71224

**Published:** 2024-01-19

**Authors:** Slimane Khayi

**Affiliations:** 1 Institut National de la Recherche Agronomique, Rabat, Macao

In the article “The genome sequence of a hoverfly, *Epistrophe eligans *(Harris, 1780)”, the authors present a near complete assembly of a female hoverfly genome sequence using a combination of  PacBio HiFi reads and Hi-C data. This resource will undoubtedly support research on the species and help elucidate an eventual impact of climate change its population dynamics.

Are sufficient details of methods and materials provided to allow replication by others?

Yes

Is the rationale for creating the dataset(s) clearly described?

Yes

Are the datasets clearly presented in a useable and accessible format?

Yes

Are the protocols appropriate and is the work technically sound?

Yes

Reviewer Expertise:

Genomics, bioinformatics, population genomics, Genomic selection, MAS, GWAS

I confirm that I have read this submission and believe that I have an appropriate level of expertise to confirm that it is of an acceptable scientific standard.
